# Genome Sequence of Bacillus cereus Strain TG1-6, a Plant-Beneficial Rhizobacterium That Is Highly Salt Tolerant

**DOI:** 10.1128/genomeA.00351-18

**Published:** 2018-05-10

**Authors:** Juan Ignacio Vílchez, Qiming Tang, Richa Kaushal, Shenglan Chen, Renyi Liu, Huiming Zhang

**Affiliations:** a Shanghai Center for Plant Stress Biology, Chinese Academy of Sciences, Shanghai, China; b CAS Center of Excellence in Molecular Plant Sciences, Chinese Academy of Sciences, Shanghai, China

## Abstract

The complete genome sequence of Bacillus cereus strain TG1-6, which is a highly salt-tolerant rhizobacterium that enhances plant tolerance to drought stress, is reported here. The sequencing process was performed based on a combination of pyrosequencing and single-molecule sequencing. The complete genome is estimated to be approximately 5.42 Mb, containing a total of 5,610 predicted protein-coding DNA sequences (CDSs).

## GENOME ANNOUNCEMENT

Bacillus cereus strain TG1-6 is a highly salt-tolerant, Gram-positive bacterium that was isolated from the rhizosphere ofSpartina anglica in Zhangpu Yanchang in Fujian Province, China. This strain has been deposited in the China General Microbiological Culture Collection Center (CGMCC) collection in Beijing under reference number 15407. Our bacterial isolations from this area have yielded a number of salt-tolerant bacteria, which mostly belong to the genera Bacillus and Pseudomonas, and which also include Arthrobacter siccitolerans strain 4J27, which was previously reported as desiccation tolerant (1, 2). At least half of the strains in this collection have been identified as producers of auxin, aminocyclopropane-1-carboxylate (ACC) deaminase, and polyamines, or as phosphate solubilizers, displaying the characteristics commonly described in plant growth-promoting bacteria that are capable of increasing plant biomass and/or stress tolerance (3–6). B. cereus TG1-6 is effective in enhancing plant tolerance to drought stress; it is also a producer of auxin and ACC deaminase and is spore forming and motile by flagella (J.I.Vílchez and H. Zhang, unpublished data).

We sequenced the complete genome of B. cereus TG1-6 by combining pyrosequencing and single-molecule sequencing. The pyrosequencing was performed with an Illumina HiSeq platform (Core Facility of Genomics, Shanghai Center for Plant Stress Biology, China), and the single-molecule sequencing was performed with a PacBio platform (Tianjin Biochip Corporation, China) (7–10). A shotgun sequencing strategy was applied to the pyrosequencing, and 14,257,603 paired reads (150 bp) were obtained. This assembly yielded 2 contigs (both greater than 500 bp), with an average length of 2,715,947 bp. The genome was completely assembled by contigs and not by scaffolds. Full sequencing resulted in a sequencing depth of approximately 262-fold. Meanwhile, the single-molecule sequencing produced 105,383 reads with a mean read length of 13,464 bp and an *N*_50_ length of 19,373 bp. The total number of sequenced bases was 1,418,902,495. For *de novo* assembly, Canu v1.5 was used with the default parameters; the genome correction step was performed by using Illumina data, with the support of Pilon v1.18 software (11, 12). This assembly yielded an average of 2,715,947 bp. The genome was completely assembled by contigs and not by scaffolds. Subsequently, the estimated genome size of 5.42 Mb was deduced from the contigs. Genes, including protein-coding DNA sequences (CDSs), were predicted by a pipeline implemented by Prokka v1.12 (13). On a whole-genome scale, the G+C content accounts for only 35.26% of the B. cereus TG1-6 genome, which was found to contain 5,610 protein-coding genes, 3 rRNA operons, and 106 tRNA genes.

With the complete genome sequence information ofB. cereus TG1-6, one can predict some biological pathways, including the biosynthesis pathways of the insecticide δ-endotoxin, antibiotic subtilin, and fungicide thiazole. Similarly, the production of flagella, spores, phenazine, and polysaccharides can also be predicted based on the genome sequence information. In addition, genome annotation also revealed the presence of genes involved in the biosynthesis of antioxidants such as glutathione, bacillithiol, riboflavin, and vitamins B12 and B8. Further investigation with this complete genome information will provide in-depth insights into the mechanisms underlying B. cereus TG1-6-induced plant stress tolerance and other potential impacts on plants, and thereby contribute to the development of B. cereus-based biotechnological applications.

### Accession number(s).

The complete genome sequence of Bacillus cereus TG1-6 has been deposited in the TBL/EMBL/GenBank databases under the accession numbers CP026678 and CP026679 and BioProject number PRJNA430755.
